# Correction: Effect of acute alcohol intoxication on mortality, coagulation, and fibrinolysis in trauma patients

**DOI:** 10.1371/journal.pone.0304895

**Published:** 2024-05-31

**Authors:** Il-Jae Wang, Byung-Kwan Bae, Young Mo Cho, Suck Ju Cho, Seok-Ran Yeom, Sang-Bong Lee, Mose Chun, Hyerim Kim, Hyung-Hoi Kim, Sun Min Lee, Up Huh, Soo Young Moon

The second affiliation for the first author is missing. Il-Jae Wang is also affiliated with #3: Department of Emergency Medicine, Pusan National University School of Medicine, Gyeongsangnam-do, Yangsan, Republic of Korea.

## References

[1] Wang I-J, Bae B-K, Cho YM, Cho SJ, Yeom S-R, Lee S-B, et al. (2021) Effect of acute alcohol intoxication on mortality, coagulation, and fibrinolysis in trauma patients. PLoS ONE 16(3): e0248810. 10.1371/journal.pone.0248810 33755680 PMC7987171

