# Supplementary material for: Effect of acute alcohol intoxication on mortality, coagulation, and fibrinolysis in trauma patients
Source: PLoS One. 2021 Mar 23;16(3):e0248810. doi: 10.1371/journal.pone.0248810 (PMC7987171; doi:10.1371/journal.pone.0248810)
Supplement: Attachment — Submitted filename: #reply1_editage_final.docx [file pone.0248810.s001.docx]

Dear reviewers,

We would like to thank and appreciate the editor and reviewers for spending their valuable time and efforts for reviewing our manuscript. The manuscript has benefitted from your insightful comments and suggestions. We have made our best efforts to address all the issues indicated in the review report, and believe that the revised version of the manuscript meets the publication requirements of the journal. Below, we have provided point-by-point responses in blue colored font to each of your comments. Revisions made in accordance to these comments have been highlighted in yellow in the revised manuscript.

Reviewer #1: The authors have performed a retrospective analysis of prospectively collected data evaluating the association of ETOH intoxication with mortality and ROTEM parameters in trauma patients. The authors show that ETOH intoxication is associated with increased clotting time, decreased lysis and increased mortality in their cohort. I have the following comments and questions for the authors:

1. What were the indications to perform ROTEMs in trauma patients during the course of the study? Essentially, what were the inclusion criteria for the study? This is a busy trauma center and over a 3 year period only 686 patients were included indicating a certain population was targeted.

> Thank you for your valuable comments. The requirement for ROTEM examination for each trauma patient is determined by the attending physician in charge based on hemodynamic signs, FAST (Focused Assessment with Sonography in Trauma) result, and injury mechanism at our trauma center. ROTEM was performed when the possibility of emergency surgery or massive transfusion was high; therefore, not all patients admitted to the trauma center underwent the procedure. We have added, “The necessity for performing ROTEM tests for each patient at our trauma center was determined by the attending physician in charge based on hemodynamic signs, FAST (Focused Assessment with Sonography in Trauma) results, and injury mechanism.” to the Methods section of the revised manuscript accordingly. Further, we have added “Fourth, the necessity performing a ROTEM test was decided by the physician based on hemodynamic sign, ultrasound results, and injury mechanism. Hence, the possibility of a selection bias cannot be totally excluded” as a limitation to the discussion section of the revised manuscript.

2. Was there a difference in mechanism of injury between the alcohol positive group and alcohol negative group? Mechanism of injury should be included in the statistical analysis because it could affect outcome. Similarly, was there a difference in AIS regions injured? The alcohol positive patients were more likely to have a low GCS, did they present more commonly with TBI which could explain all of the results of the study? To best answer this question, the authors should provide cause of death comparison between groups. The authors should discuss whether the lower GCS in the ETOH group was due to intoxication versus brain injury versus shock which could all cause a decreased GCS.

> Thank you for pointing out important aspects that may have been missed in our manuscript previously. In the revised manuscript,

1) We have additionally categorized the patients according to the mechanism of injury and then compared the effects due to the mechanism of injury between the alcohol positive and negative groups. We found that the mechanism of injury did not differ substantially between the two groups, except for driver and passenger collision, which was more frequent in the alcohol negative group.

2) We have also analyzed the data based on AIS. The proportion of AIS in head and neck, thorax, and abdomen was compared between the two groups. Interestingly, the AIS of head and neck was significantly higher in the blood alcohol positive group, but that of thorax and abdomen was not. Further, as indicated by you, the alcohol positive group consisted of higher number of patients with lower GCS scores and TBI compared to the alcohol negative group.

A description of the analysis of mechanism of injury and regional AIS has been added to the methods and results sections, and table 1 of the revised manuscript.

3) Unfortunately, there was no information regarding cause of death in our database, and the cause of low GCS in the alcohol-positive group is unclear. As the reviewer commented, consumption alcohol, head trauma, and shock can all lower the GCS scores. We found that patients in the alcohol positive group had a higher head AIS, and were in shock (lower SBP, and higher lactic acid) when compared to those in the alcohol negative group. We presume that the cause of the lower GCS in the alcohol-positive group is due to alcohol and head trauma rather than shock, but unfortunately, there is no clear evidence.

We have added, “In our study, patients in the alcohol positive group had lower GCS, lower SBP, higher head AIS and higher lactic acid compared to those in the alcohol negative group. We believe that the reason for lower GCS was not just alcohol, but also the effects of head trauma and the state of shock” to the discussion section of the revised manuscript.

3. Patients who were positive for alcohol presented with a higher lactate level. This could indicate that they presented with a higher degree of shock or it could be secondary to alcohol consumption. This requires a discussion from the authors and an opinion.

>Thank you for this insightful comment. Alcohol has the potential to induce metabolic acidosis. During the metabolism of alcohol, pyruvate is reduced to lactate, which creates lactic acidosis [*J Trauma.* 2005;58:959 –966.]. Hence, it is challenging to distinguish the cause of lactate elevation in a trauma patient who consumed alcohol, as it could be due to high degree of shock or metabolism of the alcohol.

One previous study reports that lactate is less effective as a mortality predictor in alcohol positive trauma patients (*The American journal of emergency medicine*, 2015, 33.5: 607-613.). However, further studies are required to devise methods to identify the cause of lactate elevation in such patients.

4. There is no clear association between administration of TXA and increased mortality in patients with fibrinolysis shutdown. In fact, randomized trials of TXA in trauma patients do not suggest it causes fibrinolysis shutdown. The comment about TXA should be removed from the conclusions because the authors have no data to substantiate the statement.

> We thank the reviewer for the valuable comment. Accordingly, we have removed “The administration of TXA should be considered more carefully while treating alcohol-intoxicated trauma patients” from the conclusion section of the revised manuscript.

Reviewer #2: The authors have compared ETOH, ROTEM and outcomes is a selected group of trauma patients, and correlated lab values with ETOH. I have several comments

1. the authors admit > 1000 severely injured patients a year, but over 4 years only have rotem data on 837, what was the criteria to run the rotem test?

> Thank you for your valuable comments. The necessity for performing ROTEM examinations was determined by the attending physician in charge based on hemodynamic signs, FAST (Focused Assessment with Sonography in Trauma) result, and injury mechanism at our trauma center. ROTEM test was performed when the possibility of emergency surgery or massive transfusion was high. Hence, only few among the total number of patients admitted to the trauma center underwent the procedure. We have added, “The necessity for performing ROTEM tests at our trauma center was determined by the attending physician in charge based on hemodynamic signs, FAST (Focused Assessment with Sonography in Trauma) results, and injury mechanism.” to the Methods section of the revised manuscript. Further, we have added, “Fourth, performing ROTEM test was decided by the physician based on hemodynamic sign, ultrasound results, and injury mechanism. Hence, the possibility of a selection bias cannot be totally excluded” as a Limitation to the discussion section of the revised manuscript.

2, why was the cut of value of (>10.1 mg/dL) used for etoh ?

> This cut off value was determined by the manufacturer of ETOH test kit, Roche. Below 10.1 mg/dL, the ETOH value was not recorded as a numerical data. Hence, this cutoff value intended by the manufacturer was adopted in our study to categorize patients into positive and negative groups. We have added “Blood alcohol (ethanol) was tested in the majority of severely traumatic patients by using Roche diagnostics instruments and reagents (Rotkreuz, Switzerland).” to the method section of the revised manuscript. Further, we have added, “This cutoff was determined by the manufacturer, Roche diagnostics and values of ETOH below 10.1 mg/dL were not recorded as a numerical data. Hence, this cutoff value intended by the manufacturer was adopted in our study to categorize patients into positive and negative groups” to the result section of the revised manuscript.

3. what was the criteria used for measuring ETOH?

> There was no definite criteria used for measuring ETOH, but majority of the severely traumatic patients were subject to ETOH testing. It is assumed that ETOH tests were not conducted if too much time had passed since the occurrence of injury based on the attending physician’s judgement. We have added, “Blood alcohol tests were not conducted if too much time had passed since the occurrence of the injury based on the attending physician’s judgement“. to the methods section of the revised manuscript.

4. when were the samples drawn?

> As described in the Data collection and variables section, blood samples were drawn within 15 min of initial presentation to the trauma center. The sentence, “For ROTEM and laboratory analysis, blood samples were drawn within 15 min of initial presentation to the trauma center. “ has been highlighted in yellow in the method section of the revised manuscript.

5. the authors definition of MT, > 10 units of RBCs in 24 hrs, is outdated and perpetuates the serious issue of survival bias. Especially as this is a study of coagulation Please use a more modern definition, eg 3 units in 6 hrs or something similar.

> Thank you for the valuable comment. Accordingly, we applied a modern definition of MT with reference to Cantle, Prediction of massive transfusion in trauma. [Crit Care Clinics. 2017;33(1):71-84]. Since we obtained transfusion data which was within 4 hours of the trauma, we used the ≥ 5 units RBCs within 4 hrs rule to define ‘substantial bleeding’ and applied it to the revised analysis; since the frequency of new MT (≥10 units in 6 hrs) in that reference was a severe cutoff for our population. Therefore, by this standard, the frequency of substantial bleeding was significantly higher in the alcohol positive group. The definition of substantial bleeding and the results of the revised analysis have been added to the methods, results, and tables of the revised manuscript.

6. the real issue here, is there an association between ETH and more bleeding, rather than just abnormal lab values. The association between etoh and lab values has been shown many times.

> Thank you for your valuable comment. However, it is challenging to comment on the association between ETH and increased bleeding. It is quite difficult to estimate the amount of bleeding quantitatively, by any means other than lab values and transfusion record. Thanks to the reviewers' comments in question 5, we were able to apply the concept of substantial bleeding to our research. According to the concept of substantial bleeding, we found that patients in the alcohol positive group received more RBCs in 4 hours compared to those in the alcohol negative group, which indicates an association between ETOH and increased bleeding.

7. the authors associate ETOH and mortality, but what was the cause of death in the patients and when did they die? this issue is really important as the authors have an opportunity to tease out this important issue, as they do see an association between etoh use, rotem and mortality.

> Thank you for the valuable comment. Regretfully, the final cause of death is not systematically recorded in the Korean Trauma Database, as described in the response of the second question.

We compared time from injury to death between the alcohol positive and negative groups. It was relatively shorter in the alcohol positive group than that in the alcohol negative group (54hr vs. 34 hr, p=0.056). Overall, patients in the alcohol positive group were more injured, transfused, and consequentially showed higher mortality rates compared to those in the alcohol negative group.

Since the time from injury to death is shorter among patients in the alcohol positive group, we suspect that bleeding and neurological injury were common causes of death than multi organ failure in the patients of this group. In general, massive bleeding causes death faster than that due to multi organ failure.

However, some studies have reported that death is associated with multi organ failure and hyperfibrinolysis is associated with bleeding [J Trauma Acute Care Surg. 2014;77: 811-817], it is considered valuable to identify whether similar results occur in alcohol intoxicated trauma patients. Unfortunately, the exact cause of death could not obtained from the current database. Hence, further studies with data on the cause of death are warranted.

We have added information about the time to death in the methods and results section of the revised manuscript.

8. How many patients and in what groups was TXA used, and was it given before the ROTEM sample collected?

> Thank you for the valuable comment. We apologize that there was no information regarding the use of TXA in our database, and hence, we could not access the efficacy of TXA in our study sample. In our region, TXA was not used in pre-hospital stage and ROTEM samples were collected before TXA was administered. We have added “ROTEM samples were collected before tranexamic acid was administered” to the methods section of the revised manuscript.
